# Macrophage-like Cells Are Responsive to Titania Nanotube Intertube Spacing—An In Vitro Study

**DOI:** 10.3390/ijms23073558

**Published:** 2022-03-24

**Authors:** Madalina Georgiana Necula, Anca Mazare, Andreea Mariana Negrescu, Valentina Mitran, Selda Ozkan, Roxana Trusca, Jung Park, Patrik Schmuki, Anisoara Cimpean

**Affiliations:** 1 Department of Biochemistry and Molecular Biology, Faculty of Biology, University of Bucharest, 91-95 Splaiul Independentei, 050095 Bucharest, Romania; necula.madalina92@gmail.com (M.G.N.); andreea.mariana.negrescu@drd.unibuc.ro (A.M.N.); valentina.mitran@bio.unibuc.ro (V.M.); 2 Department of Materials Science WW4-LKO, Friedrich-Alexander University, 91058 Erlangen, Germany; anca.mazare@fau.de (A.M.); so53@st-andrews.ac.uk (S.O.); schmuki@ww.uni-erlangen.de (P.S.); 3 Advanced Institute for Materials Research (AIMR), National University Corporation Tohoku University (TU), Sendai 980-8577, Japan; 4 Faculty of Engineering in Foreign Languages, University Politehnica of Bucharest, 313 Splaiul Indendentei, 060042 Bucharest, Romania; truscaroxana@yahoo.com; 5 Department of Pediatrics, Division of Molecular Pediatrics, University Hospital Erlangen, 91054 Erlangen, Germany; jung.park@uk-erlangen.de; 6 Regional Centre of Advanced Technologies and Materials, 78371 Olomouc, Czech Republic; 7 Department of Chemistry, Faculty of Science, King Abdulaziz University, P.O. Box 80203, Jeddah 21569, Saudi Arabia

**Keywords:** macrophage, inflammation, TiO_2_ nanotubes, intertube spacing, cytokines, osteoclastogenesis

## Abstract

With the introduction of a new interdisciplinary field, osteoimmunology, today, it is well acknowledged that biomaterial-induced inflammation is modulated by immune cells, primarily macrophages, and can be controlled by nanotopographical cues. Recent studies have investigated the effect of surface properties in modulating the immune reaction, and literature data indicate that various surface cues can dictate both the immune response and bone tissue repair. In this context, the purpose of the present study was to investigate the effects of titanium dioxide nanotube (TNT) interspacing on the response of the macrophage-like cell line RAW 264.7. The cells were maintained in contact with the surfaces of flat titanium (Ti) and anodic TNTs with an intertube spacing of 20 nm (TNT20) and 80 nm (TNT80), under standard or pro-inflammatory conditions. The results revealed that nanotube interspacing can influence macrophage response in terms of cell survival and proliferation, cellular morphology and polarization, cytokine/chemokine expression, and foreign body reaction. While the nanostructured topography did not tune the macrophages’ differentiation into osteoclasts, this behavior was significantly reduced as compared to flat Ti surface. Overall, this study provides a new insight into how nanotubes’ morphological features, particularly intertube spacing, could affect macrophage behavior.

## 1. Introduction

In the last decades, titanium (Ti) and its alloys have been widely used as standard materials for various implantable devices due to their outstanding properties such as high biocompatibility, low Young modulus, good tensile strength and flexibility, and an enhanced corrosion resistance in comparison to other metallic materials such as stainless steel and cobalt-chrome (Co-Cr) alloys [1,2].

However, despite their exceptional success as orthopedic implantable biomaterials, their osseointegration ability is often insufficient for a proper bone-implant integration, leading in time to mechanical instability and, ultimately, to implant failure [3]. To address these drawbacks, current research efforts have been focused on altering surface properties of Ti implants through various chemical and physical methods [4]. However, regardless of the already established impact of certain physico-chemical characteristics of Ti surfaces, such as roughness, wettability, porosity, etc., on bone forming cells adhesion, proliferation, differentiation, and subsequent new bone formation [2,5], little is known about the effects generated by these altered surfaces on the immune cells’ activity [6]. Simultaneously, altering the surface properties and varying its functionalization, excessive inflammation and formation of fibrous tissue can be avoided [7]. To fully address the implant osseointegration capacity, the response of both bone cells and macrophages should be investigated and considered when designing new implants [8].

To date, amongst various strategies of surface modification, electrochemical anodization stands out due to its cost-effectiveness, simplicity, and ability to fabricate nanostructures with tunable and controllable characteristics [9,10,11,12]. By altering the anodization parameters (voltage, time, current, and electrolyte composition), numerous anodic TiO_2_ nanostructures can be fabricated on Ti supports, in particular, nanotubes [2,9,13], nanopores [14,15,16], nanochannels [17,18,19,20,21], nanocylinders [22], or nanopillars [23]. Different manufacturing methods also lead to additional nanostructures such as nanodots [24] or nanofibers [3]. Each of these exhibits a unique set of physico-chemical and mechanical properties, affecting their biological performance. Further surface properties changes will induce a wide range of physico-chemical and mechanical signals [25,26,27], which the immune cells will pick up and translate into biological responses. Thus, any nanotopographical feature modification will reflect in the functional cellular response and in the microimmune environment generated by the inflammatory cells. Most probably, the cellular mechanism responsible for this different response is governed by the protein adsorption profile, an initial event responsible for inducing morphological and molecular changes at a cellular level [28]. The presence of monocytes/macrophages at the implantation site plays an important role in the implant’s biological performance, by promoting either its integration or rejection [29]. Extensive studies have investigated the nanotopography’s influence on macrophage activation and its role in triggering the host’s response [30,31,32,33,34].

Recent studies on anodic TiO_2_ nanotubes (TNTs) have shown promising results, both in vitro and in vivo, regarding bone regeneration and immune cellular response. For example, TNTs with diameters of 70–80 nm were capable of inducing a phenotype switch towards a M2 macrophage polarization state with an enhanced expression of specific anti-inflammatory markers, while 30–40 nm diameter TNTs were associated with high levels of pro-inflammatory cytokine release (M1 phenotype) [35]. Furthermore, Shen et al. [36] demonstrated that 110 nm diameter TNTs could increase the early inflammatory response of RAW 264.7 macrophages by activating integrin/FAK-mediated MAPK and NFκB signals and by simultaneously promoting gene expression of chemotactic mediators, as compared to 30 nm nanotubes and Ti substrates. Wang et al. [8] reported that Ti implants can be endowed in vitro with osteoimmunomodulatory properties through macrophage polarization regulation via implant surface modification with different diameter TNTs. Namely, 80–100 nm diameter TNTs induced a switch towards a pro-inflammatory M1 state, while smaller diameter (30 nm) TNTs displayed anti-inflammatory effects. Additionally, a smaller TNT could benefit macrophage cell adhesion and proliferation, while a larger one could reduce the inflammatory response [37].

Another study by Rajyalakashmi et al. [38] hypothesized that using different surface roughness and surface feature size on anodized Ti can result in better control over the macrophage inflammatory response. The in vitro results showed that after 24 h in culture, a reduced density of macrophages could be observed on nanotextured and nanotubular Ti supports compared to conventional Ti samples. Chamberlain et al. [39] explored the in vitro inflammatory response of macrophages to different diameter TNTs (30, 50, 70, and 100 nm), with the 70 nm diameter TNTs being the most advantageous support in terms of eliciting the weakest inflammatory response, compared to the commercially available Ti surface. In contrast, Sun et al. [40] demonstrated that RAW 264.7 cells presented a better adhesion and survival rate on 30 nm and 70 nm diameter nanotubes, compared with 120 nm TNTs. Moreover, 120 nm TNTs provided a stimulatory effect in terms of bone morphogenic protein (BMP)-2 and transforming growth factor (TGF)-β expression compared with smaller diameter TNTs. 

Additionally, a more recent study by Gao et al. [41] revealed that superhydrophilic 100 nm diameter TNTs (obtained by hydrogenating the anodic TNTs) can modulate the inflammatory activity of RAW 264.7 cells. Such TNTs displayed the ability to reduce the proliferation rate and activate the macrophages towards an anti-inflammatory phenotype under standard conditions, while lipopolysaccharide (LPS) stimulation decreased the pro-inflammatory response in terms of cytokine expression. Moreover, the superhydrophilic surface upregulated the gene expression of M2 surface markers and downregulated the pro-inflammatory M1 specific markers. In contrast, the superhydrophilic TNTs of Ma et al. [42] and their in vivo data revealed that smaller diameter (30 nm) TNTs presented a higher degree of bone formation, coupled with a reduced inflammatory process and a lower number of infiltrating macrophages when compared to 80 nm TNTs and polished Ti surfaces. Additionally, the 30 nm TNTs induced a pro-healing M2 polarization, both in vitro and in vivo, while the 80 nm TNTs promoted a pro-inflammatory M1 phenotype. 

In this context, the intrinsic immunomodulatory effects of nanomaterials are not only essential to assess the in vitro biocompatibility but also to determine the implant’s fate in terms of osseointegration. This is increasingly difficult as the available literature data show contradictory results in relation to the biological effects generated by TNT materials. For example, some studies suggested that smaller nanotube diameters (<70 nm) can modulate the macrophage activity towards an anti-inflammatory state [8,36,42], while others indicated that larger diameter TNTs (≥70 nm) could lessen the inflammatory reaction [35,37,39,41,43]. Therefore, it is essential to ascertain how the TNT characteristics influence the immune cells response. Most of these studies focus on the influence of nanotube diameter on the immune response, while other potentially equally important morphological (inner-tube eccentricity and intertube spacing) and spatial (geometric arrangement) parameters are yet to be approached [44]. Hence, the intertube (tube-to-tube) spacing is an important parameter which could affect the initial cellular adhesion and implicitly the entire inflammatory cellular response. onsidering that the nanotube spacing influence on the immune cells is not well documented, the present study aims to compare the immune response of RAW 264.7 cells maintained in contact with anodic TiO_2_ nanotubular surfaces exhibiting a lateral spacing of 20 nm (TNT20) and 80 nm (TNT80) under standard culture conditions or in the presence of a pro-inflammatory stimulus represented by bacterial LPS. Both TNT structures had an inner diameter of ≈78–80 nm, this choice being motivated by our previous studies which indicated a higher ability for the 78 nm diameter TNT to attenuate the in vitro inflammatory activity of RAW 264.7 macrophages compared to flat Ti [43,45]. In a more recent work [46], we showed that nanotubes with intertube spacing of 18 nm and 80 nm exert differential effects on the in vitro behavior of preosteoblasts. 

## 2. Results and Discussions

### 2.1. Nanotube Morphology and Characterization

**Figure 1:**
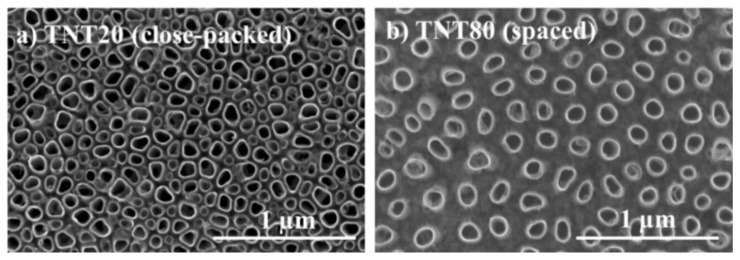
Top view SEM images showing the morphology of the nanotubes with two different intertube spacings: (**a**) TNT20, and (**b**) TNT80.

Anodic TiO_2_ nanotubes, i.e., grown by electrochemical anodization, typically have a close-packed configuration, with up to 30 nm intertube spacing at the top of the nanotubes (namely, the tube-to-tube spacing which is also directly proportional to the nanotube diameter) [9]. We have previously shown that nanotubes with distinct intertube spacing throughout the tube length, can be obtained in specific conditions, e.g., in electrolytes containing diethylene glycol, dimethyl sulfoxide, or triethylene glycol electrolytes [13,47,48,49,50]. Based on the previously established critical parameters that control the intertube spacing of nanotubes grown in diethylene glycol-based electrolytes (water content, fluoride ions amount, anodization temperature, etc.) [13,51], a specific control over the tube spacing was achieved [46]. This enabled the growth of nanotubular structures with a fixed diameter (≈80 nm) but with different spacing, 20 nm—TNT20—or 80 nm—TNT80—(Figure 1), while also having a similar tube length. The obtained nanostructures showed a good long range order, were amorphous, and presented overall a similar chemical composition [46] (TNT80 show a slightly higher fluorine content due to the anodization electrolyte, but the nanotopographical cues are a dominant factor with regards to cell adhesion and proliferation, compared to the fluorine content [52]). In addition, contact angle measurements indicated a hydrophilic behavior for Ti foil with a contact angle of 42.5°, and superhydrophobic for both TNT20 and TNT80 with contact angle values of 3° or <3°.

### 2.2. Macrophage Adhesion, Morphology, and Cell Proliferation 

A weakness of synthetic biomaterials that target biological functionality (increasing, replacing, or restoring) is represented by the immune reaction they evoke in the human body, which can compromise cells needed for bone tissue regeneration. This hinders the natural wound healing process and the successful integration of the implant [53]. While surface modification has become an instrumental tool in improving the implant’s biocompatibility and osseointegration [54], many coatings elicit little to no significant effect on the innate immune cell’s activity [55,56]. Recently, reports showed that surfaces which mimic the in vivo natural hierarchy of bone tissue could modulate important cellular and physiological processes such as cell adhesion [57], proliferation [58], differentiation [59,60], cytokine secretion [31,42], and macrophage fusion [61]. Moreover, many studies highlighted that the surface topography effect on the immune cells’ activity is even more complex, suggesting that not only the surface chemistry, but both size and shape of the scaffold could play a significant role in the host immune reaction [34]. Cells recognize, adhere, and respond sensitively to changes in the chemical and physical features of their immediate surroundings via their integrin receptors. Thus, cells constantly change their shape, motility, and function through an active remodeling of their cytoskeleton and focal adhesion assembly. Different from low motility cells that rely primarily on a dynamic reorganization of their actin cytoskeleton in order to adhere to a substrate, high motility cells, such as macrophages, do not possess stress fibers, but count on focal complexes and podosomes to fulfill their functions [62].

**Figure 2:**
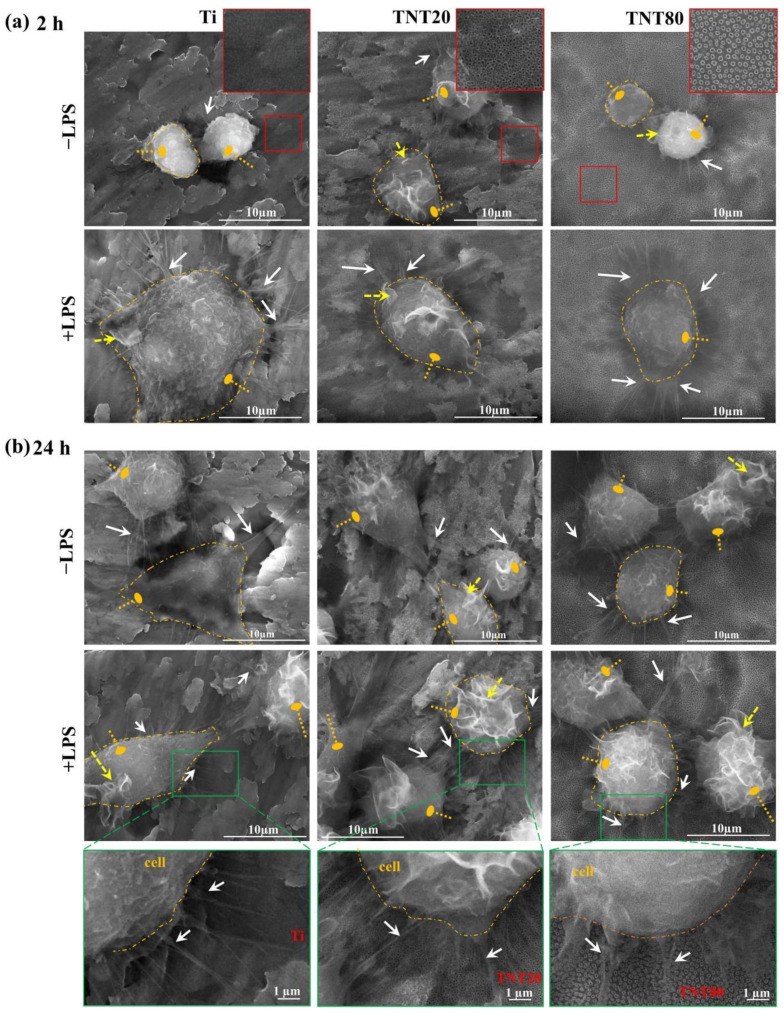
SEM micrographs of the RAW 263.7 macrophages maintained in contact with Ti, TNT20, and TNT80 surfaces for (**a**) 2 h and (**b**) 24 h, under standard (−LPS) and pro-inflammatory (+LPS) culture conditions (for each condition, a cell is delineated with dashed orange lines; oval arrow—cells 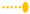; white arrow—filopodia 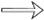; dashed yellow arrow—membrane ruffling 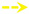). Insets in (**a**), marked red squares, show the morphology of the corresponding substrate. In the higher magnification images of the area marked with green squares for 24 h, +LPS condition (**b**), the cell membrane is delineated (orange dashed line) and the filopodia (white arrows) are indicated.

Therefore, the RAW 264.7 cells’ ability to adhere to the studied substrates was examined by SEM at 2 h and 24 h post-seeding. After 2 h of culture under standard conditions (−LPS) (Figure 2a), the images highlighted a uniform population of cells with a typical round morphology on all of the tested substrates (Ti, TNT20, TNT80). In contrast, under LPS stimulation (Figure 2a), the macrophages were characterized by larger dimensions and visible cytoplasmic protrusions, which were more numerous and thinner on the flat Ti and TNT80 (as also indicated with white arrows on the images). Additionally, cells on the Ti substrate presented a higher degree of spreading and larger dimensions (clearly evident when comparing Figure 2a +LPS left panel image with the middle and right panel images), which could indicate the presence of a strong pro-inflammatory M1 phenotype [63].

Furthermore, this morphological behavior was also observed at 24 h post-seeding when, additionally, macrophages on Ti surface exhibited an enlarged elongated shape (Figure 2b, left panels in −LPS and +LPS, see cell shape indicated by the dashed lines). On the other hand, the RAW 264.7 cells grown in contact with the nanostructured surfaces maintained their typical round shape with visible cytoplasmic protrusions (Figure 2b middle and right panels in −LPS and +LPS). This could be due to the fact that the cell’s attachment can be affected by several surface characteristics (size, shape) of the nanotopographical patterns, as well as its chemical composition [64]. Moreover, surface nanotopography can direct cells towards a more natural, spread-out morphology by altering their mechanical properties and cytoskeleton organization [65]. Our findings are in accordance with the general notion that activated macrophages display a vast array of morphological, functional, and metabolic changes compared to normal, non-activated cells [39]. Namely, they tend to present a larger cellular body and have a more pronounced ruffling of the plasma membrane, with an increased number of pseudopods and, thus, a higher capacity of spreading and adhesion [39], see also the indicated membrane ruffling in Figure 2 (dashed arrow). Recent studies have shown that these morphological characteristics are more often associated with flat Ti supports, while on nanostructured surfaces, the cells tend to extend their pseudopods through the nanostructures [66].

**Figure 3:**
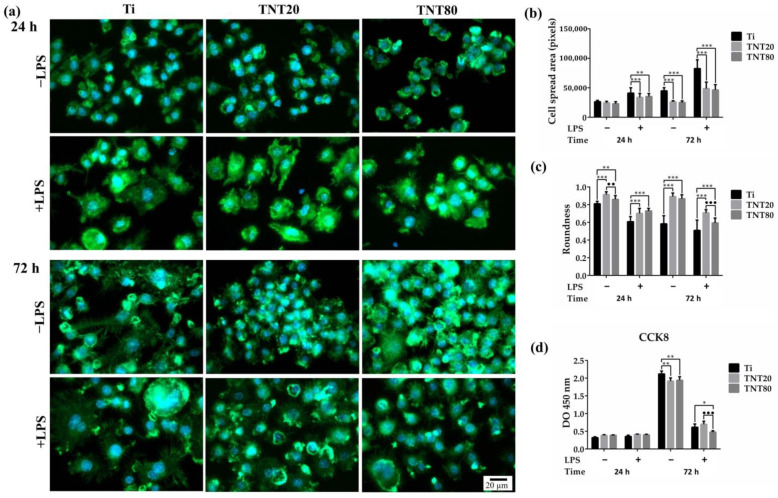
The morphological behavior and proliferation rate of RAW 264.7 cells maintained in contact with the tested surfaces for 24 h and 72 h, under standard (−LPS) and pro-inflammatory conditions (+LPS). (**a**) Macrophage morphology was evaluated by actin staining with phalloidin coupled with Alexa Fluor 488 (green fluorescence) which revealed differences in the actin cytoskeleton organization and morphological alterations under LPS stimulation. Nuclei were counterstained with DAPI (blue fluorescence). All images were captured at the same magnification (scale bar represents 20 µm). (**b**) Quantification of cell roundness. Results are expressed as means ± SD (*n* = 30 cells, ** *p* < 0.01, *** *p* < 0.001 vs. Ti, •• *p* < 0.01, ••• *p* < 0.001 vs. TNT20). (**c**) Quantification of cell spread area. Results are presented as means ± SD (*n* = 30 cells, ** *p* < 0.05, *** *p* < 0.001 vs. Ti). (**d**) RAW 264.7 cells proliferation as assessed by the CCK-8 assay (*n* = 3, mean ± SD, * *p* < 0.05, ** *p* < 0.01 vs. Ti, ••• *p* < 0.001 vs. TNT20).

As cell spreading and morphology are controlled by cytoskeleton arrangement, phalloidin conjugated with AlexaFluor 488 was used to stain the actin filaments (Figure 3a). The fluorescence microscopy images acquired at 24 h post-seeding showed that in standard culture conditions, the macrophages presented predominantly a round shape, distinctive for unstimulated cells (Figure 3a) while under LPS treatment (Figure 3a), numerous filopodia and a high degree of spreading could be observed on all of the three surfaces, with more rounded cells found on the nanotubular surfaces, as previously reported [43]. A morphometric semi-quantitative analysis further corroborated these morphologic differences (Figure 3b). Moreover, after 72 h in culture, the macrophages grown in contact with the nanostructured surfaces exhibited more evident morphologic differences in comparison with the cells on the flat Ti support (Figure 3a, 72 h middle and right panels for TNT20 and TNT80 compared to left panels for Ti). Thus, on the Ti substrate, the cells became larger, some elongated, and the number of filopodia increased, Figure 3a–c suggesting a switch in the macrophage polarization state towards a pro-inflammatory M1 phenotype, especially under LPS stimulation. On the nanotubular surfaces, no significant differences were observed, except for the cells’ roundness, which exhibited higher values for cells grown on TNT20 for 24 h in standard conditions and for 72 h under LPS stimulation (Figure 3c). 

The CCK-8 assay (Figure 3d) was used to examine the potential of the tested substrates to sustain cellular survival and proliferation. Increasing OD values were recorded with increasing incubation time for all of the investigated substrates, suggesting their capacity to support cell proliferation. Moreover, after 72 h of culture, a decrease in the number of metabolically active viable cells occurred on the nanostructured surfaces compared to flat Ti, except for the macrophages grown on TNT80 under LPS-stimulation. The prolonged treatment with LPS led to a decreased proliferation rate of the RAW 264.7 cells, and this phenomenon was previously attributed to the inhibitory effect of LPS on cell proliferation [67].

Overall, our findings are consistent with previously reported data regarding the nanotubes’ ability to tune the in vitro behavior of immune cells, particularly macrophages. For example, Tan et al. [68] studied the effects of TNTs on the capacity of RAW 264.7 macrophages to proliferate, migrate, and secrete inflammatory cytokines, concluding that such surfaces exerted inhibitory effects on these cellular parameters in standard culture conditions. Conversely, LPS loading onto the TNT substrates induced macrophage elongation with increased secretion of pro-inflammatory cytokines at 24 h post-seeding followed by a significant decrease after 72 h. Hence, it was demonstrated that the LPS-modified TNT surfaces are more potent than flat Ti with regard to modulating the inflammatory response and promoting tissue remodeling within 72 h. These results are in line with our previous data [43] showing that RAW 264.7 cells are less sensitive to LPS-induced activation on TiO_2_ nanotubular surfaces than on flat Ti, with respect to morphological behavior, gene expression, and extracellular secretion of the pro-inflammatory cytokines/chemokines and nitric oxide (NO). Moreover, Smith et al. [69] evaluated the short- and long-term immune response towards TNTs, both under standard and pro-inflammatory culture conditions, and showed a reduction in the adhesion, proliferation, and cytoskeleton reorganization for TNT surfaces. Moreover, the MTT assay and fluorescence microscopy images evidenced a lower number of cells on TNT surface compared to Ti. Altogether, these findings indicate reduced immune cell functionality on nanotubes compared to the Ti surface. 

### 2.3. Macrophage Polarization 

**Figure 4:**
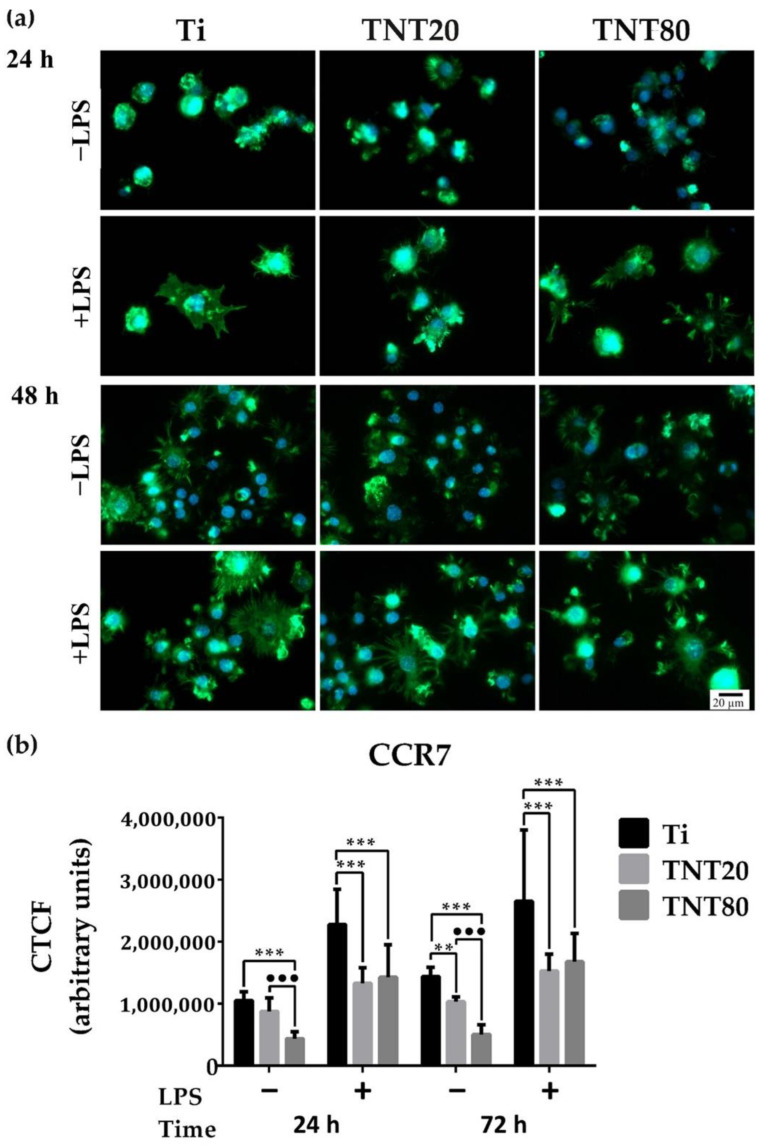
CCR7 expression in RAW 264.7 cells maintained in contact with the Ti, TNT20, and TNT80 surfaces for 24 h and 48 h, both under standard (−LPS) and pro-inflammatory (+LPS) culture conditions. (**a**) Immunofluorescence staining of CCR7 (green fluorescence: pro-inflammatory biomarker CCR7; blue fluorescence: cell nuclei). All images were captured at the same magnification (scale bare represents 20 µm). (**b**) Corrected total cellular florescence (CTCF) of individual cells quantified by using the Image J software. The macrophages grown on both nanotubular surfaces, especially TNT80, expressed significant reduction of the CCR7 fluorescence signals as compared to cells in contact with Ti substrate. Results are represented as means ± SD (*n* = 30, ** *p* < 0.01, *** *p* < 0.001 vs. Ti, ••• *p* < 0.001 vs. TNT20).

**Figure 5:**
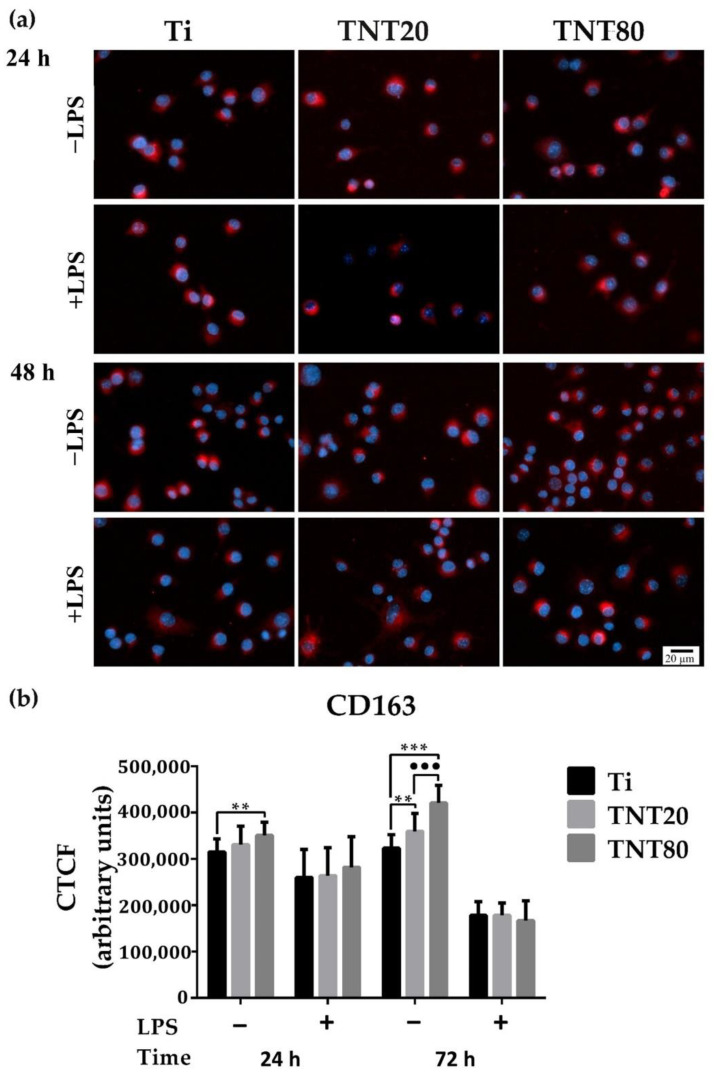
CD163 expression in RAW 264.7 cells maintained in contact with the tested surfaces for 24 h and 48 h, under standard (−LPS) and pro-inflammatory (+LPS) culture conditions. (**a**) Immunofluorescence staining of CD163 (red fluorescence: anti-inflammatory biomarker CD163; blue fluorescence: cell nuclei). All images were captured at the same magnification (scale bare represents 20 µm). (**b**) Corrected total cellular florescence (CTCF) of individual cells quantified by using the Image J software. An increase in the expression of M2 macrophage marker, CD163, was noticed on the nanostructured surfaces, mainly TNT80, when compared to the Ti sample. Results are represented as means ± SD (*n* = 30, ** *p* < 0.01, *** *p* < 0.001 vs. Ti, ••• *p* < 0.001 vs. TNT20).

To confirm whether the macrophage morphology corresponds with their phenotypic state, the protein expressions of C-C chemokine receptor type 7 (CCR7) (M1 marker) and Cluster of Differentiation 163 (CD163) (M2 marker) were evaluated by immunofluorescent staining (Figure 4a and Figure 5a). As indicated in Figure 4a, a reduction of the CCR7 fluorescence signals (green) in cells grown on both nanostructures was recorded, compared to those on Ti. Furthermore, on TNT80, the lowest levels of CCR7 were expressed, both after 24 h and 48 h of culture under standard conditions (Figure 4b), whereas the CD163 biomarker for cells cultured on TNT recorded the highest levels of expression in standard culture conditions with the most significant increase observed in macrophages grown on TNT80 (see Figure 5a for the immunofluorescent staining (red) of CD-163, and levels of expression in Figure 5b). Therefore, the TNT surfaces were capable of inducing a macrophage phenotypic switch towards an anti-inflammatory M2 state. A possible explanation may be that these nanostructures, mainly TNT80, allowed macrophages to overcome the surface spatial limitations, leading to a higher number of filopodia and implicitly to an enhanced cell adhesion. For instance, this increase in cell attachment was reported to activate actin stress fibres (RhoA)/Rho-associated protein kinase (ROCK) signalling pathway, inducing macrophage activation and sequentially their switch towards a M2 phenotype [70,71]. 

Altogether, our findings suggest that TNTs’ physicochemical characteristics can influence the macrophage polarization in a manner dependent on the intertube spacing and are consistent with a previous study showing that interrod spacing of Na_2_TiO_3_ nanorods-patterned arrays accelerated M2-polarization of RAW 264.7 cells, compared to Ti substrate [71]. Moreover, the immunofluorescence staining of CCR7 and CD163 biomarkers showed that the macrophage polarization process is time dependent [72], and at 12 h post-seeding on Sr-doped nanorods-arrays, a higher number of pro-inflammatory RAW 264.7 macrophages was observed while at 24 h, a macrophage switch towards an anti-inflammatory M2 state that persisted up to 7 days was noticed. Notably, after 72 h, the flat Ti support presented on its surface M2 macrophages, but their number was significantly lower at each experimental time point compared to that of the nanorods-arrays. However, the polarization mechanism of macrophages is more complex than initially considered, and even if literature data identified specific markers for each macrophage phenotype, the macrophages’ ability to quite rapidly adapt to any microenvironmental alteration through a switch in their polarization state can hamper their indisputable identification. Moreover, a recent study has demonstrated that the polarization process does not happen only one way and that both M1 and M2 macrophages could simultaneously contain the CCR7 and CD163 antigens [73].

### 2.4. Foreign Body Giant Cell Formation

To gain more insight into the biomaterials’ influence on the inflammatory activity of RAW 264.7 cells and into the relationship between the morphological behavior and the degree of macrophage activation, further investigations targeted the potential of the studied substrates to induce the formation of foreign body giant cells (FBGCs) from macrophage fusion. This process is unique to the macrophage phenotype [74] and occurs in various pathological conditions, e.g., including the in vivo introduction of a synthetic biomaterial. Available data reveal that synthetic biomaterials are capable of generating microenvironmental cues that can modulate the activity of various immune cells, therefore, the induced foreign body response (FBR) can be influenced by different characteristics such as surface chemistry and various physical features, i.e., topography, size, shape, and support stiffness [75]. 

**Figure 6:**
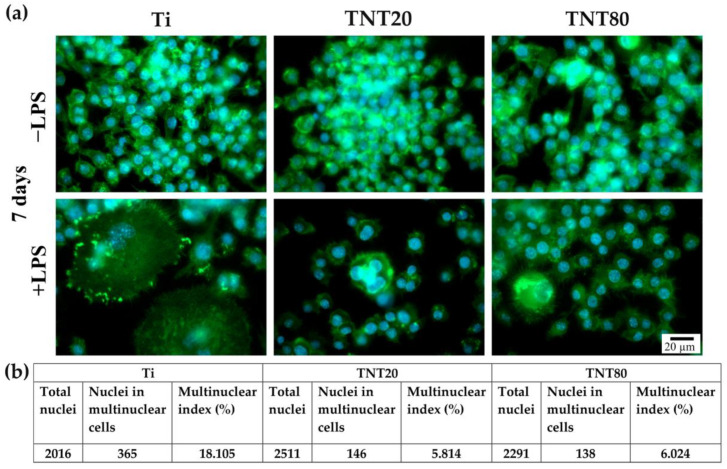
Fluorescent images of multinucleated FBGCs in direct contact with the tested surfaces at 7 days post-seeding: (**a**) Comparative fluorescent microscopy images of non-stimulated (−LPS) and stimulated (treatment with 100 ng mL^−1^ LPS) cells that indicate major morphological alterations on the flat Ti support in comparison to the TNTs surfaces and differences in terms of number, size, and cytoskeleton organization of the multinucleated cells under LPS stimulation. Moreover, the unstimulated cells presented a typical round morphology characteristic to this cell line without exhibiting cell fusion phenomenon. Cells were labeled with phalloidin coupled with Alexa Flour 488 (green fluorescence) and the nuclei were stained in blue with DAPI. All images were captured at the same magnification (scale bar represents 20 µm). (**b**) The “multinuclearity index” was calculated by examining 15–17 suggestive microscopical fields-of-view for each sample.

After 7 days of incubation, the RAW 264.7 cells presented different morphologic features depending on the investigated surface (Figure 6). Under LPS stimulation, the macrophages seeded on flat Ti (Figure 6a +LPS, left panel) exhibited characteristics specific to multinucleated giant cells, with a larger cellular body and plasma membrane displaying numerous filopodia, whereas both TNT supports generated smaller FBGCs with only 3 to 4 nuclei (Figure 6a +LPS, middle and right panel), consistent with previous results on similar close-packed TNT [43]. Furthermore, the cells grown on both TNT nanostructures showed an almost three-fold lower incidence of multinucleated FBGCs than on the Ti support (Figure 6b). This suggests that, in a pro-inflammatory microenvironment, the TNT surface could induce macrophage fusion to a lesser extent than the bare Ti surface. Notably, no significant differences in the morphological cell behavior and multinuclear index occurred between the two different TNT nanostructures. Therefore, when implanted in vivo, these surfaces could prevail FBGCs’ encapsulation and fibrous tissue growth. Additionally, in LPS absence, the RAW 264.7 cells presented a typical round morphology.

Our present findings and previously reported data [43] are consistent with those of Smith et al. [69] regarding the TNTs’ capacity to induce FBGC formation after 7 days in culture, where the TNTs were not able to induce FBGCs formation and generate an advanced immune response (as compared to the flat Ti support). Similarly, Yao et al. [37] showed that under LPS stimulation, cells grown on flat Ti exhibited the tendency to form FBGCs at 6 days post-seeding, while on TNT, they retained their typical oval morphology, characteristic for non-stimulated cells. While it is well established that the presence of FBGCs at the implantation site can cause chronic inflammation and impaired new bone formation [75,76], recent studies have reported contradictory results, where some FBGCs can express M2 markers. These results turn these cells into essential tools for the osseointegration process of non-degradable biomaterials, as well as for the degradation and replacement of degradable materials [77,78]. However, data available in literature regarding the nanotube-induced formation of FBGCs are scarce.

### 2.5. Cytokine Expression 

An important cause of local bone loss and implant failure is the generation of an improper inflammatory response, which is mainly induced by macrophage activation triggered by non-infectious material debris [79]. Due to their inert nature, once implanted in vivo, biomaterials can induce a cascade of immune responses, regulating the outcome of the integration process and the implant’s biological performance [80]. Similar to the natural wound healing process, the biomaterial-mediated immune response occurs in four stages, namely hemostatic, inflammatory, proliferative, and remodeling stage [81]. The switch from the inflammatory to the proliferative stage has been extensively studied in numerous tissue models, and literature data showed that at this point, a failed transition most often leads to an improper regenerative process. During this transitional phase, immune cells, such as macrophages and neutrophils, orchestrate effective natural healing by altering their phenotype and recruiting cells that will follow in the proliferative stage [82]. During the inflammatory stage, macrophages play a key role in determining the implants success by recognizing and adhering to the biomaterial’s surface, as well as through the secretion of cytokines, chemokines, and growth factors involved in the bone formation and remodeling processes [83]. Therefore, an improper macrophage activation can lead to excessive secretion of pro-inflammatory mediators and to a chronic immune reaction which results in an inadequate osseointegration and inflammation [84]. In contrast, a proper reaction to implantation will lead to the secretion of anti-inflammatory cytokines, which is reported to be beneficial for bone healing and implant performance [56,85].

**Figure 7:**
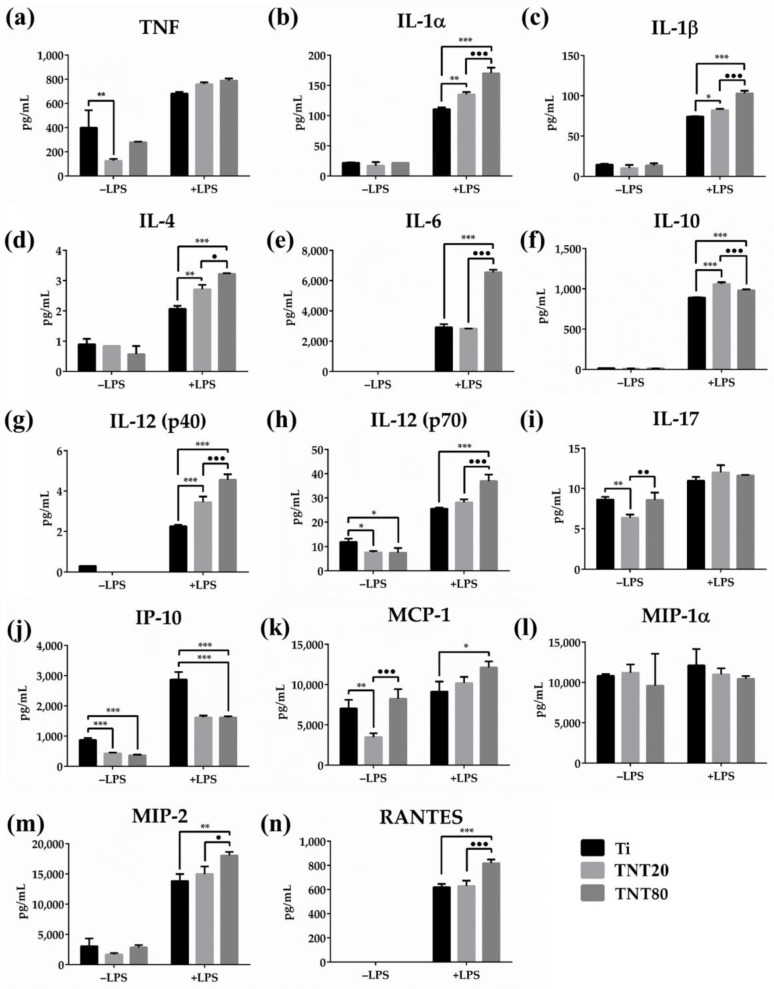
Extracellular cytokine/chemokine levels determined by Luminex-based multiplex assay, analyzed after 48 h of direct contact with the tested materials, both in standard and pro-inflammatory conditions (treatment with 100 ng mL^−1^ LPS) for (**a**) TNF, (**b**) IL-1α, (**c**) IL-1β, (**d**) IL-4, (**e**) IL-6, (**f**) IL-10, (**g**) IL-12(p40), (**h**) IL-12(p70), (**i**) IL-17, (**j**) IP-10, (**k**) MCP-1, (**l**) MIP-1α, (**m**) MIP-2, (**n**) RANTES. The highest level of cytokines/chemokines was secreted in pro-inflammatory conditions by the RAW 264.7 cells grown onto the surface of the TNTs supports, while in the absence of the pro-inflammatory agent (−LPS), very low amounts of cytokines were noticed in the cell growth media. Results are expressed as means ± SD (*n* = 3, * *p* < 0.05, ** *p* < 0.01, *** *p* < 0.001 vs. Ti; • *p* < 0.05, •• *p* < 0.01, ••• *p* < 0.001 vs. TNT20).

Cytokines play a key role in various aspects of cell growth, differentiation, and activation, and exert effects on the immune cells, and are involved in the inflammatory response evolution through a complex network of interactions [86]. Pro-inflammatory cytokines such as interleukin (IL)-6, tumor necrosis factor (TNF), and interferon (IFN)-γ are involved in the early phase of tissue healing and activation of the adaptive immune system [87], while anti-inflammatory cytokines such as IL-10 modulate the pro-inflammatory cytokine secretion through interactions with different inhibitors and soluble receptors to regulate the immune response [88]. In this context, the protein release of TNF, IL-1α, IL-1β, IL-4, IL-6, IL-10, IL-12(p40), IL-12(p70), and IL-17 was assessed by performing a Luminex-based multiplex assay. Figure 7 shows the protein expression of these cytokines at 48 h post seeding, both in standard and pro-inflammatory conditions. In standard conditions, TNTs induced an inhibition of the cytokine release in comparison to Ti. In a microenvironment that mimics a bacterial infection (treatment with LPS), as expected, macrophages secreted higher amounts of cytokines than the unstimulated cells. Moreover, the cytokine release profiles of the LPS-stimulated cells in contact with TNT80 recorded the highest values in comparison to Ti and TNT20 substrates. Overall, the cytokines’ secretion levels increased in the following order: Ti < TNT20 < TNT80. 

In the early stages of inflammation and bone regeneration, at the implantation site, the main immune mediators that create the regenerative milieu are predominantly represented by pro-inflammatory cytokines such as IL-6, TNF-α, IL-1, etc. [89,90]. Activated macrophages secrete large quantities of TNF-α and Il-6 at the fracture/implantation site within the first 24 h being involved primarily in mesenchymal stem cell (MSC) recruitment and differentiation [91]. Moreover, IL-6 was reported to enhance macrophage polarization and commitment towards a M2 phenotype [92]. Additionally, in vivo studies: (i) on an IL-6 knockout mouse model demonstrated an improper bone callus formation, a reduced number of osteoclasts, and a delayed bone healing [93], and (ii) on mice evidenced that low doses of TNF-α administered at the fracture site enhanced bone healing and highlighted the TNF-α role in macrophage polarization [94]. Furthermore, both cytokines help establish and control the homeostasis of anti-inflammatory cytokines, a key process for bone formation guidance and remodeling [95]. Similarly, IL-1β plays a key role in the early inflammatory phase and new bone formation by inducing the direct MSCs differentiation into bone-forming cells [96], and its production and secretion can be enhanced by various surface characteristics (e.g., roughness) [97]. Ainslie et al. [66] showed that on TNT, IL-1β protein expression was significantly higher than on flat Ti. Furthermore, in vivo studies on rabbit models demonstrated that IL-1β inhibition led to a reduction in bone formation, while in vitro studies demonstrated that this cytokine could stimulate cartilage explants to express the osteogenic protein (OP)-1, therefore demonstrating its powerful orthotropic activity [98]. In contrast to their pro-inflammatory character, IL-12(p40) and IL-12(p70) exhibit an essential and independent dual role during the initiation of the inflammatory response [99], with reports of their involvement in bone formation through the inhibition of the mouse receptor activator of nuclear factor Kappa B recombinant protein (RANKL)-induced osteoclastogenesis [96]. Similarly, the IL-17 early expression during the initial inflammatory response was shown to promote MSCs’ proliferation and differentiation [100,101,102], while in vivo studies on IL-17 knockout mouse models demonstrated delayed formation of the bone callus coupled with a reduced bone mineral density attributed to a low number of osteoblasts [100]. 

Considering our results and the abovementioned literature, we hypothesize that an early acute inflammatory response is essential for a proper healing of the traumatized bone tissue and for an adequate implant integration. Figure 7d,f depicts the expression profiles of the anti-inflammatory cytokines IL-4 and IL-10. Under standard conditions, they were secreted in very low amounts without no significant differences between the surfaces. However, after LPS stimulation, both TNTs exhibited significantly higher capacity to stimulate IL-10 and IL-4 production when compared to flat Ti. It is well known that IL-10 is capable of limiting the inflammatory activity of macrophages through the inhibition of certain pro-inflammatory cytokines such as TNF, IL-1β, IL-6, and IL-12 [103]. Fiorentino et al. [104] reported that in LPS-stimulated macrophages, the IL-6, IL-1, and TNF production was reduced after IL-10 treatment, while Takakura et al. [105] demonstrated that IL-10 deficient mice showed an enhanced Th1 response and were susceptible to developing LPS hypersensitivity. Moreover, Tao et al. [106] observed increased levels of IL-10 in LPS-stimulated RAW 264.7, suggesting that an enhanced production of the anti-inflammatory cytokine plays a regulatory role on cells after the pro-inflammatory reaction. Taking this into consideration, we assume that the overexpression of the anti-inflammatory cytokines, such as IL-10, by the LPS-stimulated macrophages occurs in order to ensure a timely attenuation of the inflammation.

Likewise, the release of chemokines such as IP-10 (CXCL10), monocyte chemoattractant protein (MCP)-1, macrophage inflammatory protein (MIP)-1α, MIP-2, and regulated upon activation normal T expressed and secreted protein (RANTES) was assessed (Figure 7j–n). 

Under standard conditions, the MCP-1 was produced by macrophages in significantly lower amounts on TNT20 compared to the other two substrates, while in LPS-presence TNTs, especially TNT80, an increase in the release of this chemokine was induced (Figure 7k). Likewise, in the pro-inflammatory conditions, the macrophages grown on TNT80 secreted higher levels of RANTES and MIP-2 than on flat Ti and TNT20, while in LPS absence, these levels were much lower and similar for all substrates. In the case of MIP-1α, the expressed secretion profiles were relatively similar under both experimental conditions. Note that the secretion profiles of MCP-1, MIP-1α, and RANTES recorded the same trend on Ti and TNT20 samples as in our previous work [39], suggesting that these surfaces elicit almost equivalent chemotactic activities. Regarding the IP-10 or CXCL10 (C-X-C motif chemokine ligand 10), under both culture conditions, the Ti support (Figure 7j) could induce higher levels of secretion compared to TNTs that exhibited almost equal values. 

### 2.6. Osteoclast Differentiation

**Figure 8:**
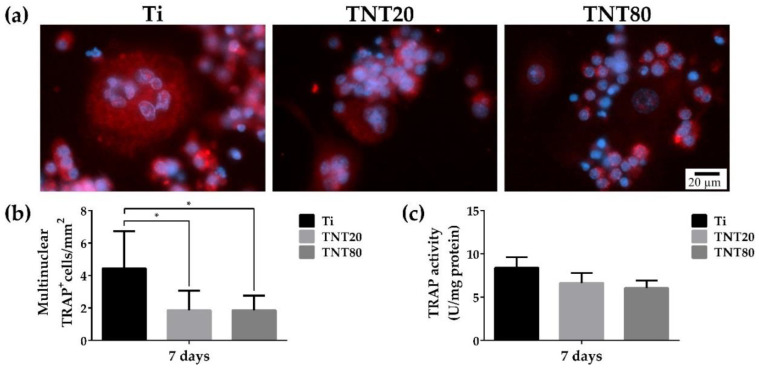
Evaluation of the tested surfaces on the osteoclastogenic differentiation: (**a**) The immunofluorescence staining of the intracellular TRAP protein revealed differences between the tested materials with the flat Ti support leading to the formation of larger TRAP-positive multinucleated cells (more than 3 nuclei), while on the TNT surfaces a more reduced number and small multinucleated TRAP cells could be observed. The TRAP signals are represented by red fluorescence, while the nuclei were stained with DAPI (blue fluorescence). All images were captured at the same magnification (scale bar represents 20 µm). (**b**) The average number of TRAP positive cell per mm^2^. Results are expressed as means ± SD (*n* = 7, * *p* < 0.05 vs. Ti). (**c**) TRAP activity determination. Results are expressed as mean ± SD (*n* = 3, no statistical differences were observed).

Due to the particular relevance of osteoclasts for bone tissue engineering, the effects of the different investigated substrates on the osteoclastogenic differentiation were investigated in terms of tartrate-resistant acid phosphatase (TRAP) protein expression by means of immunofluorescence staining. The evaluation was performed at 7 days post-seeding, when the RANKL-stimulated RAW 264.7 cells were expected to exhibit visible features characteristic for fully differentiated osteoclasts. TRAP is an intracellular enzyme involved in the osteoclast resorption activity, being widely used in various studies as an important marker for osteoclast differentiation. Our results showed that on Ti, larger TRAP-positive multinucleated cells were present, while on both TNT nanostructures, fewer and much smaller multinucleated cells could be observed (Figure 8a). The formation of large multinuclear mature osteoclasts represents an important event in osteoclast activity [107], and their size is directly correlated to their bone resorption ability. Our findings suggest that a nanotubular topography can overcome the differentiation of macrophages into mature osteoclasts. Moreover, the quantification of the TRAP-positive multinucleated cells indicated a statistically significantly lower number of mature osteoclasts on both TNTs compared to Ti (Figure 8b). However, no significant differences between the two different TNT nanostructures were remarked. Additionally, for a more in-depth analysis of the effect of these surfaces on the osteoclastogenic differentiation, the intracellular TRAP activity was investigated (Figure 8c). RAW 264.7 cells grown on TNTs exhibited lower levels of TRAP enzymatic activity compared to those on Ti, but without statistically significant differences between the different substrates. Altogether, these data indicate that the assayed nanotube interspacing does not affect the osteoclast in vitro behaviour.

Despite the important role played by osteoclasts in the osseointegration process of various implantable devices, little effort has been devoted to highlighting the influence of different metallic surface characteristics on osteoclastogenesis with more than often controversial and sometimes contradictory results. However, our results are in line with data reported by Ion et al. [19] showing that nanochannel-coated Ti50Zr surfaces were capable of inhibiting RANKL-mediated osteoclastogenesis when compared to control TiZr supports. Similarly, Lai et al. [108] reported that compared to TNT, Ti presented a higher number of TRAP-positive multinucleated cells, suggesting that TNTs could inhibit the osteoclast maturation to a greater extent.

## 3. Materials and Methods

### 3.1. Nanotube Synthesis and Characterization

The nanotubular structures were obtained on 0.125 mm thick Ti foil (99.6% pure temper annealed, ADVENT, Oxford, UK). The Ti foils were cut into 2.5 × 2.5 cm and cleaned by successive ultrasonication (acetone, ethanol, water) and dried in a N_2_ stream. 

The anodization experiments (two-electrode configuration) were performed in optimized conditions [46], briefly close-packed nanotubes with 20 nm intertube spacing (TNT20)—Glycerol (>99.7% p.a. Roth, Karlsruhe, Germany):H_2_O (70:30 vol.%) + 0.5 wt.% NH_4_F (>98% p.a. Roth, Karlsruhe, Germany), 20 V, and spaced nanotubes with 80 nm intertube spacing (TNT80)—Diethylene glycol (>99.5% p.a. Roth, Karlsruhe, Germany) + 4 wt.% HF (HF 40%, Sigma Aldrich, Germany) + 0.3 wt.% NH_4_F + 7 wt.% H_2_O, 27 V, 4 h, 30 °C. For additional information, see experimental details in previous work [46].

The morphology of the samples was evaluated by scanning electron microscope (SEM, FE-SEM 4800SEM, Hitachi, Japan). The contact-angle measurements were performed by a contact angle measurement system (DSA100, Kruss, Germany), as described previously [109].

### 3.2. Cell Culture

The RAW 264.7 macrophages (ATCC^®®^ TIB-71™, American Type Culture Collection, Manassas, VA, USA) were seeded in triplicates on the sterile surfaces of the commercial pure Ti, TNT20, and TNT80 substrates, at different cell densities, depending on the experimental approach, and incubated in standard and pro-inflammatory (treatment with 100 ng mL^−1^ LPS from *Escherichia coli*) culture conditions, as shown in our previous paper [43].

### 3.3. Cell Survival and Proliferation Assays 

The proliferation rate of the RAW 264.7 cells was evaluated at 24 h and 72 h after seeding by using a Cell Counting Kit-8 (CCK-8, Sigma-Aldrich Co., St. Louis, MO, USA) as we previously described [110].

### 3.4. Cell Adhesion and Morphology Assesment 

To assess the degree of macrophage spreading and cell morphology the actin cytoskeleton was fluorescently labeled, as previously reported [111]. In summary, after 24 h and 72 h in culture, the RAW 264.7 cells were fixed with a cold solution of 4% paraformaldehyde (PFA), permeabilized, and blocked with a solution containing 2% bovine serum albumin (BSA) and 0.1% Triton X-100. Afterwards, successive incubations with phalloidin coupled with Alexa Fluor 488 (20 µg mL^−1^, Invitrogen, Eugene, OR, USA) and 4′-6-diamidino-2-phenylindole (DAPI, Sigma-Aldrich Co., Steinheim, Germany) were performed. Subsequently, cells were visualized with an Olympus IX71 (Olympus, Tokyo, Japan) inverted fluorescence microscope and representative fields were captured using a Cell F image acquisition system (Version 5.0). Furthermore, shape parameters such as area and roundness of 30 individual cells per condition were quantified using the Image J software (Version 1.53c, National Institutes of Health, Bethesda, MD, USA). Roundness, which is defined as (4π × (cell area)/(cell perimeter)^2^), gets a value between 0 (highly elongated) to 1 (perfect round). 

In addition, the morphologic macrophage features and the cell–biomaterial interactions were evaluated by means of field-emission SEM. Thus, RAW 264.7 cells grown on the samples’ surfaces were fixed with 2.5% glutaraldehyde for 1 h and then dehydrated in a graded series of ethanol (35%, 50%, 75%, 90%, and 100%) for 10 min each. Finally, the samples were incubated with hexamethyldisilane for 10 min and air dried. The images were captured using a Quanta Inspect F50 scanning electron microscope (FEI-Philips).

### 3.5. Expression of Markers Characteristic of M1/M2 Phenotypes

An immunofluorescence assay was used to reveal the expression of M1 and M2 cell surface markers, i.e., CCR7 and CD163. Briefly, RAW 264.7 cells were seeded on the surface of the analyzed samples at an initial density of 1 × 10^4^ cells·cm^−^^2^, under both standard and pro-inflammatory conditions, for 24 h and 48 h. For each time point, the seeded cells were fixed, permeabilized, and blocked according to the protocol already described [111]. Subsequently, the cell layer was stained with anti-CCR7 antibody (1:100 dilution) (Abcam, Cambridge, UK) and anti-CD163 antibody (1:50 dilution) (Santa Cruz Biotechnology, Dallas, TX, USA). After three washes with PBS, the samples were incubated for 1 h at room temperature with AlexaFluor 488- and AlexaFluor 546-conjugated secondary antibodies, respectively. Finally, they were counterstained with DAPI and visualized in fluorescence microscopy (Olympus IX71, Olympus, Tokyo, Japan). The corrected total cell fluorescence (CTFC) was measured from 10 fields chosen randomly per sample to quantify M1 and M2 polarization markers. To calculate CTFC, the ImageJ software was used (Version 1.53c, National Institutes of Health, Bethesda, MD, USA). Therefore, to measure the area, the mean fluorescence and the integrated density, the freehand selection tool was used to manually contour each cell. The corrected total cellular fluorescence was determined by applying the following formula: CTFC = integrated density − (area of selected cell × mean fluorescence of background readings), and the result was then equalized against the mean CTFC of the neighboring interphase cells from the same field.

### 3.6. In Vitro Macrophage Fusion Assay

To assess the potential of the tested surfaces to induce FBGC formation by macrophage fusion, RAW 264.7 cells were seeded at an initial density of 2 × 10^3^ ·cm^−2^ and incubated for 7 days under both experimental conditions with the culture medium change at every 2 days. Then, the samples were processed as previously described [43] and the “multinuclear index” represented the percentage of nuclei in multinuclear cells exhibiting at least 3 nuclei relative to the total number of nuclei from a chosen field.

### 3.7. Luminex-Based Cytokine/Chemokine Detection and Quantification

Protein levels of inflammatory mediators secreted by macrophages into the culture media were measured, after 48 h of culture, with a multiplex kit (MCYTOMAG-70K-PMX MILLIPLEX MAP Mouse Cytokine/Chemokine Magnetic Bead Panel-Premixed 25 Plex-Immunology Multiplex Assay) according to the manufacturer’s instructions (Merck KGaA, Darmstadt, Germany). In the end, the concentration of each cytokine/chemokine was calculated by using a standard curve.

### 3.8. Osteoclast Differentiation Assay

To evaluate the influence of the tested materials on the osteoclastogenic process, RAW 264.7 cells were seeded at an initial density of 1 × 10^4^ cells·cm^−2^ and maintained in the culture medium supplemented with 50 ng·mL^−1^ RANKL for 7 days. At 7 days post-seeding, the expression of intracellular TRAFP protein was evaluated by immunofluorescent staining as previously reported [111]. Multinuclear TRAP-positive cells were counted in 10 random fields and data were presented as the number of TRAP^+^ cells/mm^2^. The cells containing at least three nuclei were identified as mature osteoclasts. To have a more complete view of the osteoclastogenic process, the TRAP intracellular activity was investigated, as previously reported [19].

### 3.9. Statistical Analysis

Statistical analysis of data was performed with GraphPad Prism software (Version 6, GraphPad, San Diego, CA, USA) using one way/two-way ANOVA with Tukey’s multiple comparison tests. All values are expressed as means ± standard deviation (SD) and differences at *p* < 0.05 were considered statistically significant.

## 4. Conclusions

The results presented in this study demonstrate that with respect to the anodic TiO_2_ nanotubes, the nanotube’s intertube spacing can influence, to a different degree, the macrophage in vitro response in terms of cell proliferation, actin cytoskeleton organization, cell fusion, and inflammatory cytokine/chemokine secretion. Therefore, designing nanotubular structures with well-defined nanotube interspacing can provide a potential passive approach for macrophage modulation with the purpose of obtaining an immune response directed towards bone healing and implant osseointegration rather than chronic inflammation and bone resorption.

## Data Availability

Not applicable.
